# Towards a Situated Spatial Epidemiology of Violence: A Placially-Informed Geospatial Analysis of Homicide in Alagoas, Brazil

**DOI:** 10.3390/ijerph17249283

**Published:** 2020-12-11

**Authors:** Blake Byron Walker, Cléssio Moura de Souza, Enrique Pedroso, Ryan S. Lai, Paige Hunter, Jessy Tam, Isaac Cave, David Swanlund, Kevan Guilherme Nóbrega Barbosa

**Affiliations:** 1 Institüt für Geographie, Friedrich-Alexander-Universität Erlangen-Nürnberg, 91058 Erlangen, Germany; clessio.moura.souza@fau.de; 2 Department of Geography, Simon Fraser University, Burnaby, BC V5A 1S6, Canada; enrique_pedroso@sfu.ca (E.P.); ryan_lai_2@sfu.ca (R.S.L.); paige_hunter@sfu.ca (P.H.); jessyt@sfu.ca (J.T.); isaac_cave@sfu.ca (I.C.); david_swanlund@sfu.ca (D.S.); 3 Department for the Professional Master Programme in Health Research, Campus IV, Centro Universitário CESMAC, Macieó 57051-530, Brazil; kevanguilherme@gmail.com

**Keywords:** Brazil, GIS, crime, homicide, violence, social determinants, infrastructure, spatial epidemiology

## Abstract

This paper presents an empirically grounded call for a more nuanced engagement and situatedness with placial characteristics within a spatial epidemiology frame. By using qualitative data collected through interviews and observation to parameterise standard and spatial regression models, and through a critical interpretation of their results, we present initial inroads for a situated spatial epidemiology and an analytical framework for health/medical geographers to iteratively engage with data, modelling, and the context of both the subject and process of analysis. In this study, we explore the socioeconomic factors that influence homicide rates in the Brazilian state of Alagoas from a critical public health perspective. Informed by field observation and interviews with 24 youths in low-income neighbourhoods and prisons in Alagoas, we derive and critically reflect on three regression models to predict municipal homicide rates from 2016–2020. The model results indicate significant effects for the male population, persons without elementary school completion, households with reported income, divorced persons, households without piped water, and persons working outside their home municipality. These results are situated in the broader socioeconomic context, trajectories, and cycles of inequality in the study area and underscore the need for integrative and contextually engaged mixed method study design in spatial epidemiology.

## 1. Introduction

### 1.1. Rationale

Immense increases in computational capability, data availability, methodological sophistication, and ease-of-implementation have facilitated rapid growth in the number of studies under the broad umbrella of applied spatial epidemiology. Studies range from mapping and visually evaluating point patterns of disease to geographical correlation studies and multivariate analysis using complex Bayesian spatiotemporal lag models and spatially aware artificial intelligence [1]. The development and release of increasingly user-friendly Geographic Information Systems (GIS) with sophisticated modelling capability, whether via private enterprise (e.g., ArcGIS) or open source (e.g., GeoDA, QGIS/SAGA) platforms, has made it possible for non-experts to quickly create geostatistically complex disease maps with only a few mouse clicks, without a fundamental understanding of, and critical reflection on, the limitations of a dataset, the assumptions of a given model, and the sociopolitical contexts defining and influencing both the phenomenon/relation of interest and the processes of quantitative analysis and cartography. Rather than serving as a reminder of these critiques from previous studies, this study attempts to underscore the importance of contextualisation in spatial epidemiology through an applied case study of homicide in Alagoas, Brazil.

In contrast to epidemiology as a global/public health practice, in which individual case-level analysis, fieldwork, and social, political, economic, and cultural contexts play a significant role, spatial epidemiology within academic spheres tends to direct more focus towards the analyst-level. Concretely, this tendency has positioned the practice of applied spatial epidemiology research as a spatial variant of public health epidemiology [1], whereby the data, methods, and quantitative/cartographic results constitute the core of a given study, and context and relevance are assessed at the (sub-)population scale. That detailed consideration of local sociopolitical/cultural contexts and individual lived experiences/local knowledge tends to be secondary, if not entirely absent, is likely due to several factors: (i) the ecological fallacy and its spatial progeny, the modifiable areal unit problem, both of which essentially prohibit individual-level prediction based on sample/population-level analyses; (ii) study programmes centred on GIS/geostatistics generally do not feature any qualitative methodological training; (iii) structural factors defining efficient academic practice such as disciplinarity, word count limits, and a tendency towards favouring quantity in career-related assessments (e.g., publication counts and citation indices). These observations are not new. Since the early 1990s, critical voices predominantly embedded in contemporary human geography and Geographic Information Science have persistently made calls for a greater degree of contextual and qualitative engagement in the use of GIS and spatial analysis [2]. This line of argumentation has crystallised perhaps most prominently around theoretical interrogations of a quantitative–qualitative dichotomy defining medical and health geographies; in brief, the former draws upon predominantly statistical methodologies rooted in a positivist or deterministic epistemology, whilst the latter tends to leverage pluralist or constructivist approaches lending themselves to a greater focus on individuals’ lived experiences of health and illness and their embeddedness in sociopolitical structures and relations [3]. Efforts to intertwine or hybridise both conceptualisations of the space/place–health relation have largely stemmed from researchers positioned in sectors of GIScience predominantly concerned with social theory and its neighbouring foci, resulting in a substantial theoretical literature and a number of notable studies using GIS or spatial analysis to complement a primarily qualitative analysis [4]. However, for over two decades it has been consistently argued both outside and within the parameters of peer review that medical geography/spatial epidemiology has yet to sufficiently engage with non-positivist epistemologies and interdisciplinary methodology [5].

Drawing on previous efforts to integrate qualitatively/critically-informed conceptualisations of place in spatial epidemiological analysis [6,7,8], this study seeks to make a further increment towards the instantiation of a “situated spatial epidemiology”, defined here as placially-informed geospatial analysis of health/disease, in which (a) local and embedded contextual knowledge support both the modelling itself and the interpretation of its results, and (b) the position of the researcher/analyst is actively reflected upon throughout the research process. While the following empirical study itself does not constitute a holistic analysis, as it is limited by both limited scope and a lack of detailed precedent, it does serve as an initial and exploratory increment, through which the authors seek to critically assess the feasibility of this approach and reflect on future prospects for a situated spatial epidemiology in contemporary health and medical geographies. As a test case, we leverage qualitative data for the parameterisation, interpretation, and contextualisation of explanatory (spatial) regression models to assess municipal-scale socioeconomic risk factors for homicide rates in the Brazilian state of Alagoas.

### 1.2. Empirical Context

In 1996, the World Health Organisation passed Resolution WHA-49.25, in which interpersonal violence was framed as a leading public health problem worldwide. The most recent United Nations Global Study on Homicide estimates that 465,000 persons were victims of homicide in the year 2017, of which 64,078 were reported in Brazil alone [9]. At the subnational scale, the state of Alagoas ranks among the most violent federal states [10], with 1811 reported homicides in 2017 alone: a crude homicide rate of approximately 55 per 100,000 persons, higher than that of any country worldwide [9,11,12].

The majority of homicides in Alagoas occur in the capital city of Maceió, one of 102 municipalities in the state. The structure of the city uniquely features many wealthy neighbourhoods directly adjacent to impoverished/less affluent areas, known as favelas (unplanned hillside settlements), grotas (unplanned settlements in valleys and along drainage canals, a term unique to Macieó), deprived bairros (un/planned urban neighbourhoods), and conjuntos habitacionais (planned social housing projects in the form of large peripheral suburbs) [13]. Favelas, grotas, deprived bairros, and conjuntos habitacionais feature high concentrations of drug trafficking and other forms of crime due to several geographical factors: they are located near urban centres and wealthy neighbourhoods, feature a complex layout of narrow streets, a dense built environment, and an irregular topography, and contain large populations of youths from low-income households.

The physical infrastructure of informal settlements is often insufficient for the needs of residents. The buildings are multifunctional in design in order to meet both occupational and domestic needs [14], but they often do not have legal or reliable access to running water and sanitation; this lack of access is even more common in rural areas throughout the state of Alagoas, and while Brazil’s National Sanitation Plan was terminated in the 1980s, there have been no effective policies or programmes to address this issue [15]. As such, access to running water and sanitation remains a strong proxy for socioeconomic status in Alagoas [16].

The relationship between socioeconomic deprivation and violence has been intensively studied in a health/medical geographical context in recent decades, with the wide majority of analyses indicating a strong positive covariance between socioeconomic deprivation and interpersonal violence, i.e., that counts and rates of violence tend to be higher in more deprived countries [9], regions (WHO violence report), and neighbourhoods [17]. The consistency of this relationship across geographical scales underscores its stability and highlights the utility of socioeconomic deprivation both for modelling/predicting violence and for developing policy to curb it.

In the Brazilian context, socioeconomic conditions are known to play a significant role in violence incidence [7]. Socioeconomic divisions stemming from a long and tumultuous colonial history (Over 40% of African persons sold in the colonial slave trade landed in Brazil, where their labour was exploited predominantly for the production of sugarcane, precious minerals, and ranching until 1888 [18]) have been exacerbated by rapid economic growth in the last half-century, the benefits of which are predominantly experienced amongst middle and higher socioeconomic classes, leading to further socioeconomic disparities [19]. This process creates conditions where racially, economically, socially, geographically, or otherwise marginalised youths are susceptible to participation in violent activities [9]. Past studies indicate that a likely mechanism for this effect stems from frustration amongst low-income individuals who live in proximity to wealth, who are then more likely to engage in high-risk activities to attain wealth, such as robbery or drug trafficking [20,21]. Socioeconomic deprivation therefore constitutes an important constellation of variables for analysing and building a nuanced understanding of the drivers of violence in regions with high inequality.

In previous spatial epidemiological studies of violence, it has been demonstrated that income measures, (un)employment, and educational attainment can function as useful predictor variables [17,22], including in the Brazilian context [23], as they operate as proxies for the intensity and nature of local labour markets and their linkages to violence and space [24]. In the context of our study area, the rapid urbanisation of the population in recent decades has led to the immense growth of Alagoas’ favelas, grotas, and conjuntos habitacionais, as rural migrant workers tend to settle in these areas due to financial circumstances (i.e., low opportunity in rural areas serves as a push factor while affordability in deprived urban neighbourhoods serves as an attractor [14]). However, low educational attainment in rural regions limits many internal economic migrants’ employment prospects and earning potential, effectively entrapping them in a cycle of poverty, through which informal settlements and project housing communities fail to serve as economically transitional, rather becoming permanent spaces of socioeconomic exclusion [14]. Multiple forms of social and economic marginalisation experienced by residents of these spaces present significant barriers to participation in formal economies, effectively forcing many people into informal, or sometimes illegal, forms of labour [24]. Official government estimates state that approximately 50% (the true figure is likely higher, according to local experts) of all residents in low-income neighbourhoods in Macieó are engaged in informal or illegal labour, which frequently involves drug trafficking and involvement in criminal groups, where individuals experience a high risk of involvement in interpersonal violence [13,25].

The role of educational attainment is well understood to be a vital element in the linkages between socioeconomic deprivation and violence [9]. The implications of a lack of education on violence and homicide are evident in Alagoas, where many youths from low-income households drop out of school in order to increase household income levels [26]. However, a minimum legal employment age of 16 (constitutionally raised from 14 in 1996) and a lack of education prevents their entry into formal economies, so they work in informal economies [27]. In doing so, many never complete primary or secondary education and therefore remain economically marginalised, thereby completing the cycle [13,25].

In the Brazilian context, sex/gender also appear to exhibit a significant role in violence and homicide. The observed demographic involved in violence and homicide, both as perpetrator and victim, is disproportionately young and poor, self-identifies as black and male, has little or no access to education, and inhabits spaces of extreme wealth inequality, dominance of informal economies, and a high concentration of organized crime [13,19,25,26,28].

However, Brazilian women experience very high rates of violence, often occurring within the household or from an intimate male partner. The rates of femicide in Maceió (10.7 per 100,000) are more than double the national average (4.8) [29]. These rates are hypothesised to reflect family instability, gender inequality, and local socioeconomic deprivation [21], all of which contribute to an increased risk of a family member being involved in criminal activity [13,19,25,28], which in turn correlates with women’s risk of familial and gender-based violence [29]. This cycle constitutes a cyclical environment of instability and generational trauma. However, despite a legal and societal tendency to address familial/intimate partner violence as a private issue, non-consensual divorce is becoming an increasingly accessible legal means of leaving a violent or otherwise abusive domestic environment and may serve as a means of measuring familial stability [29]. 

### 1.3. Objectives

This study features two distinct objectives: (i) to provide an initial exploration of the utility of local knowledge for informing and situating the practice of spatial epidemiological analysis in an applied research sphere, and; (ii) to test for aspatial and spatial associations between area-level socioeconomic deprivation and homicide through a public health lens.

## 2. Materials and Methods

The empirical methods for this analysis were conducted in three phases: (i) a snowball review of the studies on violence and socioeconomic deprivation was conducted and qualitative data were collected via transcribed interviews and participant/field observation; (ii) based on the results of the first phase, we selected candidate variables from the Brazilian census and acquired homicide data at the municipal scale in Alagoas. These variables were entered into an aspatial linear regression model, a spatial lag model, and a spatial error model to identify socioeconomic correlates with homicide rates, and (iii) model results were contextualised using local expert knowledge and interview/observation findings. 

### 2.1. Qualitative Data

A snowball literature review was conducted from September 2019 until April 2020, during which academic and non-academic sources in English and Portuguese were collected and assessed for their thematic suitability. A total of 171 sources were indexed. Contextual information specific to Brazil, Alagoas, interpersonal violence, homicide, and socioeconomic/built environments was recorded and categorised heuristically to derive (a) an adequate depth of background information to the point of saturation and (b) a list of candidate socioeconomic criteria suitable for modelling homicide rates in the study area.

Interview data and participant/field observation were drawn from fieldwork conducted by the second author in Alagoas during two data collection periods in 2013 and 2016, during which time the author conducted site/participant observation and 24 semi-structured interviews with local youths involved in violence and additional interviews and discussions with their family and community members, persons employed in the legal system, and other experts and community stakeholders. All participants provided informed consent and were anonymised for transcription. Data were collected in low-income communities across Macieó, in all correctional facilities for youths and adults in Macieó, and whilst travelling through the city with youths and community members. Following data collection, interviews were transcribed using 129 subject-derived codes and analysed using the QDA software package, and field observations/photographs were categorised and used to supplement and contextualise the results of the qualitative analysis. More detail on the data collection and analysis is provided by Moura de Souza [13].

### 2.2. Quantitative Data

Homicide data from January 2016 to February 2020 (inclusive) for every municipality in Alagoas (*n* = 101 during the study period) were acquired from the publicly accessible data portal of the Public Security Secretariat of the State of Alagoas (SESP). All recorded homicide records were downloaded with victim age, sex, date and time of death, municipality of residence, and cause of death (by ICD-10 group). Homicides were considered to be all cases with ICD codes X85–Y09 inclusive. The number of homicides was recalculated into an average crude rate per 100,000 life-years, based on each municipality’s number of homicides and its official estimated population for each year in the study period.

Socioeconomic data for each municipality in the 2010 Brazil Census were acquired from the publicly accessible database provided by the Brazilian Institute for Geography and Statistics (IBGE), the federal agency responsible for the decennary national census. A total of 167 census indicators across all socioeconomic categories were collected and random samples were drawn and checked for errors against figures published by IBGE to ensure data fidelity; no data for the selected indicators were missing or featured erroneous or implausible values. Variables were thematically categorised manually (e.g., income measures, education, household amenities) and each category was examined and deliberated independent of the others. The deliberation phase was conducted amongst the authorship through eleven meetings and frequent informal discussions from February to August 2020, during which each category of interest was discussed in the context of the qualitative data, and linkages to interview results were made. For example, the prevalence of legal household piped water as a means of delineating spaces of urban poverty emerged through field visits and interviews with residents of grotas and favelas in Macieó. After a further filtering stage, during which excessively precise variables were manually removed from the categories (e.g., number of persons employed in emergency medical services who earn less than 1.5 times the minimum wage), a list of 93 variables remained. In the next phase, the results of the qualitative analysis were linked to potential explanatory variables using hand-drawn mind maps and a simple Likert scale to represent suitability in Microsoft Excel, where a variable’s score out of ten represented its heuristically-assessed suitability as determined by the first two authors and the final author. The most prevalent or central variables from the mind maps and the highest-scoring variables from the Likert ranking were then independently evaluated for their suitability for modelling through further discussions in light of the results of qualitative data collection, resulting in 31 candidate explanatory variables, which were entered into preliminary linear regression models using a stepwise parameterisation to explore effects and familiarise the authors with patterns in the data. Further discussion with the two co-authors from Alagoas and the removal of variables exhibiting excessive multicollinearity resulted in a further reduction to six final predictor variables: male population, persons without elementary school completion, households with reported income, divorced persons, number of persons whose place of employment is outside their municipality of residence, and households without piped water. The selected predictors were then standardised by resident population or number of households where appropriate and mapped by municipality using the software package QGIS (v.3.10.4). Manual data validation and correction was conducted prior to modelling and no transformations were necessary.

### 2.3. Modelling

Modelling and mapping were conducted using R (v.1.3) and GeoDA (v.1.14). This study used ordinary least squares (OLS) regression to assess bivariate and multivariable linear relationships between the six selected socioeconomic predictors and crude homicide rates. Despite the relatively restrictive model assumptions compared to semiparametric approaches, we selected OLS for interpretability and conceptual simplicity in order to improve accessibility for a broader multidisciplinary audience. Bivariate and multivariable preliminary models using the qualitatively-informed selection of candidate variables were run to assess fit and multicollinearity and overfitting, and experimental dummy variables were included for the major cities of Macieó and Arapiraca, based on evidence that the unique socioeconomic, cultural, and built environment structures of these sites may exhibit differences with the remainder of the study area. Similar experimentation was conducted with population density as a standalone parameter and interaction term. These exhibited small, insignificant effect sizes and were therefore excluded from the final model. 

Model fit and residuals were visually assessed by plotting and interpreting residuals and statistically assessed using Jarque–Bera, Koenker–Basset, and Breusch–Pagan tests. The spatial autocorrelation of residuals was tested using Moran’s I with a contiguity neighbourhood definition and threshold selection. In addition to the OLS model, we ran two common variants that are able to account for spatial effects: spatial lag and spatial error. A spatial lag model is a geographical variant of OLS where independent variables are assumed to exhibit a given degree of effect on the dependent variable of neighbouring areas, thereby improving estimates that would otherwise be unreliable due to clustering effects. In contrast, a spatial error model is a geographical variant of OLS in which spatial effects are estimated using the error terms, rather than in the predictor variables themselves, thereby allowing unspecified spatial effects to be modelled; spatial error model coefficients therefore reflect unknown spatial effects. Both spatially weighted models used a first-order queen contiguity definition for their spatial weight matrices, selected heuristically based on discussions with local experts and tested in preliminary sensitivity analyses. 

## 3. Results

### 3.1. Qualitative Analysis

Field observation in low-income communities, whether planned (conjuntos habiatcionais), semi-planned (bairros), or unplanned (favelas and grotas), yielded strong similarities in the socioeconomic situations, settings, and trajectories of their residents, despite significant differences in their respective built environments. These sites feature informal markets and concentrations of open-air bocas (“mouths”), locations where illicit drugs (predominantly crack cocaine and cannabis) are sold by male youths associated with organised crime groups, known in Brazil as facções.

The youths interviewed described their experiences within the socioeconomic and legal structures of Alagoas similarly, including: an insufficient living environment, e.g., poor/no sanitation in the household, small living spaces with minimal privacy, and a dense and polluted neighbourhood environment; low access to schools due to excessive travel distance, poor quality of education, and/or classes not held due to a dearth of teaching staff (many teachers are disincentivised from working in low-income neighbourhoods due to low pay and danger, e.g., armed robbery); a lack of professional development, apprenticeships, and legal employment opportunities, and; familial factors often also play a significant role, e.g., single-mother households and weak parental control. These factors were directly linked to criminal and (potentially) violent activity, as the participants described a search for accessible means to improve their individual, family, and community socioeconomic conditions.

Only two of the twenty-four participants had any formal work experience, and many described a point at which they transitioned from informal employment to criminal means of earning. The participants indicated that the sale of ~50 g of cocaine per week would earn them double the average adult income in their community, which despite the risk of arrest or violence, enables them to financially contribute to their families and purchase socially important status symbols. A sense of excitement, belonging, and social cohesion were also cited as motivations for involvement in criminal activity.

In addition to drug trafficking, robbery emerged as a common means of acquiring wealth amongst the participants. Unwritten codes generally forbid robbery within one’s own neighbourhood, so many persons travel via public transport or motorcycle to surrounding areas to commit robbery, thereby spatially disconnecting the criminal act and site of potential violence from one’s place of residence.

### 3.2. Quantitative Analysis

A total of 6705 homicides were recorded for the study period. As shown in Table 1, the majority of homicides are a result of intentional (pre-meditated) homicide. Femicide refers specifically to homicides in which the mens rea or intent is specifically linked to the victim’s gender, comprising one-fifth of all homicides in the study period. The five reported femicides featuring a male victim have been verified against official records. This may be due to a data entry error or that the male victims may have been a secondary victim in a homicide incident in which a female victim was the primary target. Temporally, a diurnal pattern was observed in which the majority of incidents occurred between 18:00 and midnight (44%), with minor peaks between 05:00 and 07:00 (9%) and 14:00 and 16:00 (13%).

**Figure 1:**
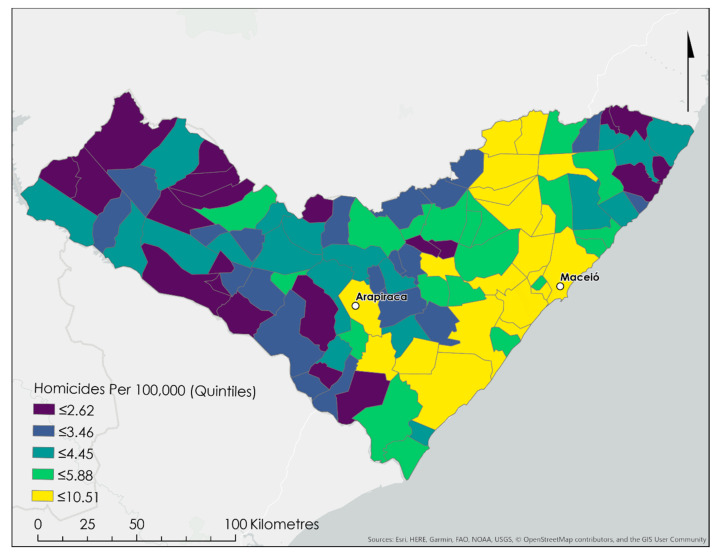
Spatial distribution of crude homicide rates in the Brazilian Federal State of Alagoas, featuring clustering of high rates in the two largest cities of Macieó and Arapiraca, along the relatively wealthy Atlantic coastal region, and in the sugarcane corridor north of Macieó.

As shown in Figure 1, homicide rates exhibit a strong east-west gradient, where areas with high rates are located in the cities of Arapiraca and Macieó, clustering along the Atlantic coast (east of Macieó), and extending to the northern border with the neighbouring state of Pernambuco. The state capital of Macieó featured a homicide rate of 271 per 100,000 person-years while the highest homicide rates were concentrated in the neighbouring municipalities of Pilar (341), Marechal Deodoro (290), and Rio Largo (270).

Model coefficients are shown in Table 2, indicating that the male proportion of the municipal population exhibits a very small positive effect on homicide rates, and while the effect associated with low educational attainment is stronger (a 1% increase in the proportion of the population without primary school completion predicts an additional 53 homicides per 100,000 life-years), the effect size for divorce rates is nearly 12 times greater. The proportion of households with reported income, households without piped water, and the percentage of workers whose place of employment is outside their home municipality were associated with decreases in homicide rates. The directionality and strength of these effects were consistent between all three models presented in Table 2, suggesting that the variable selection and parameterisation were adequately robust, with the exception of the spatial lag model coefficient for households without piped water, which exhibits a low effect size and high *p*-value.

**Figure 2:**
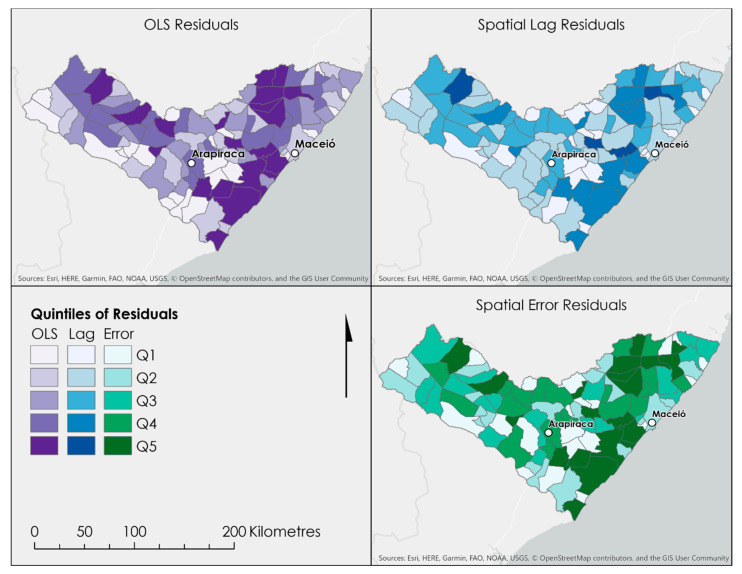
Regression model residuals indicating regions where the socioeconomic models overpredicted (higher quintiles) and underpredicted (lower quintiles) homicide rates in the study area.

All three models exhibited moderate fit (R^2^ > 0.62) and no significant multicollinearity among predictors. The OLS model residuals were normally distributed and featured a slight positive skew (Jarque–Bera (df = 2) = 8.43) and homoscedasticity (Koenker–Bassett (df = 8) = 8.46, *p* = 0.39). The spatial lag model exhibited a near-normal distribution of residuals and homoscedasticity (Breusch–Pagan (df = 8) = 9.5), and a likelihood ratio (df = 1) of 11.01 indicates some remaining spatial effects. The spatial error model also exhibited homoscedasticity (Breusch–Pagan (df = 8) = 12.96) and a likelihood ratio (df = 1) of 1.12 indicates no significant spatial effects remaining. The spatial error model showed a slight increase in performance over the aspatial OLS and spatial lag models, and similar coefficients to those in the aspatial OLS indicate robustness, while the improved model fit (R^2^ = 0.66) indicates that accounting for spatial effects may have contributed to improved parameter estimates, although this result is not confirmed in our study design. Distinct spatial clustering of residuals for all three models is evident in Figure 2 and was confirmed with Moran’s I, indicating underlying spatial effects and clustering, even in the spatial error model, which indicates persistent clustering and geographical heterogeneity of effects; potential contextual factors may explain these effects, as explored using the results of the qualitative analysis below.

## 4. Discussion

### 4.1. Spatial and Socioeconomic Context

The use of interview data, local expert knowledge, and field/participant observation facilitated a contextually informed selection of six key predictors for homicide rates at the municipal scale in Alagoas. The models were able to account for over 60% of the variance in homicide rates, but failed to account for all spatial effects, as evidenced by the persistent clustering. High-rate clusters surrounding Macieó, e.g., Pilar, Marechal Deodoro, and Rio Largo, may indicate spillover effects, such that homicides related to the Macieó drug trade are carried out in the surrounding municipalities. A detailed qualitative analysis of individual cases, including a comparison of place of residence and place of death, may be necessary to determine to what degree drug-related violence is exported from Macieó to the surrounding areas. Clusters of homicides were observed along the Atlantic coastline, a relatively wealthy corridor known worldwide for beach resorts and tourism. The residual maps indicate that the three socioeconomic models underestimated the homicide rates in these municipalities, indicating that additional factors likely account for this pattern. Based on local expert knowledge and interview data collected in Alagoas, the authors hypothesise that the high homicide rates in these areas are linked to the presence of powder cocaine markets in these areas, where a more affluent local and tourist clientele exhibits a preference for the higher-value substance, thereby creating a profitable market for facções. Conversely, clustering north of Macieó near the Pernambuco border may be related to the historical context of northern Alagoas, where sugarcane production has been concentrated since the 18th century. The sugarcane industry is deeply embedded in Brazil’s history of slavery and post-slavery racial marginalisation, which continues to exclude non-white Brazilians from formal economies to this day [30,31,32].

A lack of education in adolescence increases the difficulty of finishing school during adulthood as many youths, particularly young men, are vulnerable to involvement in criminal groups [9,28]. The municipality of Maceió features an insufficient number of education and health facilities [33], such that many youths from low-income areas have poor or no access to adequate primary education, as observed in the qualitative analysis findings. Our model results indicating a correlation between area-scale low educational attainment and homicide rates correspond to a previous United Nations study [9]. Education can not only decrease a violence and homicide rates but can also keep youths off the streets and away from dangerous lifestyles [9]. Investment in education has the potential to lower the rates of violence and homicide in Alagoas by allowing high school graduates to access jobs that would not be available without higher levels of education.

Despite femicide being explicitly enacted as a crime in 2015 (predominantly as a means of countering legal defence in honour-killing cases) and the landmark decision of the constitutional Maria da Penha Law (MPL) that established women’s support services and stricter punishment for perpetrators, the problem persists [29]. The largest barrier is that most of the services stemming from the MPL are concentrated in state capitals and access is difficult for those who live elsewhere [29]. We found a positive correlation between divorced persons and domestic violence that indicates a complex relationship between marital status, inequality, and abuse. The social norms of privacy, widespread domestic violence, and the status of women in Brazilian society make it difficult for women to escape violent situations, and this continues to influence rates of gender-based violence and homicide. Legal changes in recent decades have made it easier for victims of familial violence to obtain a divorce order. Prior to the Divorce Act in 1977, a marital union could only be dissolved through death, annulment, agreement, or litigious resolution [34]. While significant societal/religious pressures may still prevent victims from pursuing separation/divorce to escape a violent spouse [35], divorce rates have continued to rise since the mid 1980s.

The interview results demonstrated a strong association between youth violence and organised crime in Alagoas, as documented in more detail [13,26]. Criminal groups have had a large social presence in Brazil since the 1980s, when there was an arrival of large-scale cocaine trade to the country [27]. Since then, criminal groups have spread into various states and rural areas [36]. Criminal groups are typically composed of young men from the early teens to mid-twenties who are recruited from favelas, bairros, grotas, conjuntos habitacionais, and prisons [37]. Criminal groups participate in local violence when defending territory or committing robbery, and they use violence as a symbolic act to establish dominance.

Brazil is facing an increasingly serious threat from its two major criminal organizations: the First Capital Command (Primeiro Comando da Capital—PCC), and Red Command (Comando Vermelho). Factions within these groups are becoming more involved in the international drug trade and continue to operate extortion and kidnapping rings locally. These groups have become dispersed into rural areas since the 2014 World Cup and the 2016 Rio Olympics, a trend that matches with the rates of homicide. Large cities such as São Paulo and Rio de Janeiro have begun to see a decrease in homicide rates whereas lower population areas, like Alagoas, have been seeing an increase in homicide rates. This increase in Alagoas and other areas suggests that homicide is closely linked to the presence of criminal organizations and their involvement in the international drug trade. According to an eyewitness account, shoot-outs between police and members of criminal groups are common [38]. Organized criminal violence is used as a tool for geopolitical control as much as it is used for local violence. 

Studies examining police activity in Brazil have shown that police forces disproportionately target young African-Brazilian males from socioeconomically deprived areas [39]. In addition to killings by police, many police officers are also the targets of violence themselves. The cycle of revenge creates a situation where police and criminal groups escalate from a traditional law enforcement relationship to a situation resembling guerilla warfare. Police often engage in shoot-outs with criminal groups, particularly in favelas where drug trafficking is common [37], resulting in casualties on both sides. On average, police killings account for approximately 20% of homicides per year, whether police are the perpetrators or the victims [40]. There are multiple police institutions operating in Brazil, but we will focus on the forces that are directly involved in the policing of violent crime and homicide: the Polícia Civil and Polícia Militar. The Polícia Civil are responsible for criminal investigation, including the investigation of crimes already perpetrated. The Polícia Militar are responsible for uniformed patrol and crime prevention work, but also have the power to intervene in criminal investigations conducted by the Polícia Civil [40]. The Polícia Militar operate within territorial boundaries in an area that may encompass multiple cities and neighbourhoods, while the Polícia Civil operate around regional police stations that also have territorial boundaries but may not match the boundaries of the Polícia Militar [40]. Homicide counts reported by the Polícia Civil and Polícia Militar do not match, but both sides insist that their databases are accurate. This is indicative of disorganization and competition between state-level law enforcement institutions.

Corruption in Brazilian law enforcement is common, as officers often take bribes from donos, who are local leaders in the drug trade [41]. In 2017, multiple officers from Rio’s 7th Military Police Battalion were accused of accepting bribes from criminal groups in exchange for protection [42]. Many police killings go undocumented and punishment is rare [42]. The Brazilian government’s failure to properly address police brutality demonstrates widespread institutional corruption and fortifies the acceptance of police killings within these organizations.

### 4.2. Reflection and Limitations

This applied study sought to serve as an experimental interdisciplinary spatial epidemiological analysis, the process of which yields several key points for reflection and developing a research agenda. Many factors embedded in the research process itself provide challenges for moving towards a more situated and integrative spatial epidemiology; in this study, we sought to establish common ground between a diversity of academic backgrounds amongst the authorship (statistical analysis, criminology, epidemiology, critical human geography, environmental geography, and law), a wide range of professional experience (advanced undergraduate level, doctoral level, a registered lawyer, a clinical dental surgeon, and an associate professor), the multilingual nature of this study (research and project matters being conducted and discussed in Portuguese, German, and English), and varying degrees of experience with the study area, victims, perpetrators, and local stakeholders. These are common challenges that underscore the necessity for situating both the researchers and the research process itself and to report accordingly, despite a scientific preference for dissociation in quantitative science [43]. Indeed, these very challenges enabled us to develop a richer understanding of the research foci, parameterise models using local knowledge, and situate the results in the unique and dynamic contexts of the study area. For example, field observation, interviews, and local knowledge played a central role in the deliberation process, through which a large set of candidate variables were discussed with the co-author who conducted the fieldwork. This process facilitated a contextually enabled selection of explanatory variables in an informed manner, as opposed to using a purely data-driven approach such as stepwise regression or random forest modelling [44]. Therefore, the resulting models cannot boast the highest possible explanatory performance, i.e., preliminary exploratory models using Bayesian machine learning and artificial intelligence algorithms were able to achieve higher model fit based on more than 130 variables, but the results are nearly impossible to interpret and relate to the study area context. While it was determined at an early project stage that this modelling approach would yield quantitatively stronger results and be able to better account for outliers (such as the high homicide rate observed in Pilar), the interdisciplinary need for interpretability and conceptual simplicity points grants preference to more commonly-understood statistical methodologies [45].

An important limitation concerns the process by which candidate variables were selected based on the qualitative data analysis; the authors elected to conduct this step through open discussion, deliberation, and consensus amongst the authorship, rather than adopting a more methodologically strict qualitative approach. This flexibility allowed the authors’ understanding of the study area to play a role in the process, but a coding and weighted frequency analysis, for example, may have yielded similar results. Future research should compare approaches to this step in order to develop best-practice guidelines. Interestingly, several variables that were heuristically selected through group deliberation did not emerge as significant in the preliminary models after controlling for all covariates included in the final model: household income measures, health indicators, households without a phone, number of bedrooms, school attendance rate, and unemployment rate. However, the small number of municipalities (*n* = 101) may explain the instability in parameter estimates.

This study exhibits several key limitations that must be taken into consideration when examining the results. Most notably, there is a degree of subjectivity involved in the collection, coding, and translation of qualitative data, and its use for selecting contextually appropriate census variables for statistical modelling requires that nuance to be traded off for quantifiability, essentially necessitating a reduction in dimensionality. For this reason, a situated and informed reflection upon the modelling results is crucial. Additionally, the qualitative data stem predominantly from the city of Macieó, which is not representative of the entire study area. A geographically stratified sampling strategy may yield additional factors that better explain the rural context of homicide in Alagoas.

The usual limitations of area-based geographical analysis also apply, most prominently the modifiable areal unit problem (i.e., quantitative results exhibit a direct dependency on the size and configuration of spatial units and are often sensitive to changes in the geometry of these units). This is perhaps best exemplified in the case of Macieó, whose municipal boundaries extend well into the rural periphery. Therefore, the homicide and census data for Macieó do not reflect the reality of homicide and socioeconomic deprivation across its entire area, and a finer-resolution analysis would be preferred, were we able to acquire more precise homicide data.

More broadly, the use of precisely-defined census variables constrains the dimensionality of socioeconomic deprivation they represent, and prevents modelling of individual-level intersectional deprivation, e.g., as cross-tabulated variables such as number of lone-parent families with no piped water and no primary school completion. Similarly, the use of reported homicide rates to describe the broader burden of violence induces several significant limitations. Many cases of non-fatal familial/domestic/intimate partner violence are not reported, and the distribution of these incidents is likely underrepresented and dependent on local community-level sociocultural contexts. Other forms of non-lethal violence are not included in the dataset, and therefore the results may exhibit a bias towards more lethal trajectories, such as the drug trade, robbery, and inter-gang conflict.

## 5. Conclusions

This study explores the initial steps towards informing and situating an applied spatial epidemiological analysis through the use of qualitative contextual information and local knowledge. The use of interviews, field/participant observation, and local expert knowledge enabled the parameterisation of regression models that indicated significant associations between homicide rates and six selected socioeconomic variables at the municipal scale in Alagoas, Brazil. The proportion of male population, population without primary school completion, and the divorce rate were positively correlated with homicide rates, while the proportion of houses with reported income, houses without running water, and commuter populations were negatively correlated with homicide rates. 

The authors favoured a public health approach to the problem of interpersonal violence with a contextual focus on the underlying trajectories and cycles embedded in Brazil’s social systems. We found that wealth inequality, education, and criminal activity are closely intertwined and have strong ties to homicide, especially for the demographic of young men. Wealth inequality and both real and perceived lack of social mobility influence future potential for violence and homicide. Employment opportunities and economic policies that reduce wealth disparities are valuable for decreasing violence and homicide, and they should be adjusted to accommodate the higher costs of living in urban areas. This will increase equity and accessibility of opportunities and services for those who would otherwise have to turn to informal or illegal labour. Multiple sources agree that the law enforcement structures play a significant role in violence, and other current studies are presently investigating these linkages and processes in depth, with a focus on policing tactics and systemic corruption. We found that a lack of access to education is tied to inequality and criminal activity and serves as a gateway into a violent lifestyle. These factors do not directly contribute to homicide on their own, but rather act as a complex interrelated network of actants that create the social-built environment experienced in Alagoas. 

Altogether, this study evidences that poverty alone is not an adequate predictor of violence and homicide, and instead multiple socioeconomic deprivation factors and the social-built environment should be the focus of future research. We therefore assert the need for more contextually engaged and interdisciplinary methodologies in applied spatial epidemiological violence research and the importance of embedded and locally informed interpretations of modelling results when moving towards violence prevention policy.

## Figures and Tables

**Table 1:** Homicides grouped by intent for the state of Alagoas, Brazil between 2016 and 2020.

Intent	Count (Column %)	Female Victim (Column %)	Male Victim (Column %)
Premeditated	5856 (87%)	457 (71%)	5399 (89%)
Resistance	501 (7%)	28 (4%)	473 (8%)
Robbery	179 (3%)	24 (4%)	155 (3%)
Femicide	132 (2%)	127 (20%)	5 (<1%)
Bodily Injury	37 (1%)	6 (1%)	31 (1%)
Total	6705	642	6063

**Table 2:** Multivariable regression results indicating significant correlations between the selected socioeconomic indicators and homicide rates.

	OLS Model		Spatial Lag Model		Spatial Error Model	
Dependent Variable	Coefficient	*p*-Value	Coefficient	*p*-Value	Coefficient	*p*-Value
% male	0.0017	<0.0001	0.0016	<0.0001	0.0016	<0.0001
% without primary school	170.63	0.0113	136.1	0.0221	154.79	0.0168
% households with reported income	−65.26	0.0001	−59.39	<0.0001	−61.87	0.0001
% divorced persons	2019.2	0.0006	1794.23	0.0004	1841.86	0.0008
% households without piped water	−27.23	0.1119	−11.79	0.458	−24.64	0.165
% work outside home municipality	−85.4	0.0084	−42.31	0.1698	−84.21	0.0095
Model fit	R^2^ = 0.621 *p* = 0.621		R^2^ = 0.623 Log Likelihood = −459.73		R^2^ = 0.658 Log Likelihood = −453.60	
